# Ureteral orifice involvement by urothelial carcinoma: long term oncologic and functional outcomes

**DOI:** 10.1590/S1677-5538.IBJU.2017.0218

**Published:** 2017

**Authors:** Muammer Altok, Ali F. Sahin, Mehmet I. Gokce, Gokhan R. Ekin, Rauf Taner Divrik

**Affiliations:** 1 Department of Urology, MD Anderson Cancer Center, University of Texas, Houston, TX, USA;; 2 Department of Urology, Sivas Numune Hospital, Sivas, Turkey;; 3 Department of Urology, School of Medicine, Ankara University, Ankara, Turkey;; 4 Department of Urology, Tepecik Education and Research Hospital, Izmir, Turkey;; 5 Department of Urology, Private Ege City Hospital, Izmir, Turkey

**Keywords:** Urinary Bladder Neoplasm, Hydronephrosis, Therapeutics

## Abstract

**Purpose:**

Bladder cancer (BC) may involve the ureteral orifice, and the resection of the orifice has oncological and functional consequences such as development of upper tract urothelial carcinoma (UTUC), vesicoureteral reflux or ureteral stenosis. The aim of this study was to investigate the oncological and functional outcomes of the ureteral orifice resection in BC patients and determine the predictive factors for UTUC development.

**Materials and Methods:**

A total of 1359 patients diagnosed with BC, between 1992 and 2012, were reviewed retrospectively. Patients were grouped with respect to orifice resection and compared for development of UTUC, survival and functional outcomes. Kaplan-Meier method was used to compare survival outcomes. Logistic regression analysis was performed to determine predictors of UTUC development.

**Results:**

Ureteral orifice involvement was detected in 138 (10.2%) patients. The rate of synchronous (10.1% vs. 0.7%, p=0.0001) and metachronous (5.3% vs. 0.9%, p=0.0001) UTUC development was found to be higher in patients with ureteral orifice involvement. Orifice involvement and tumor stage were found to be associated with development of UTUC in the regression analysis. Overall (p=0.963) and cancer specific survival rates (p=0.629) were found to be similar. Hydronephrosis was also significantly higher in patients with orifice involved BC, due to the orifice obstruction caused by the tumor (33.3% vs. 13.9%, p<0.05).

**Conclusions:**

BC with ureteral orifice involvement has significantly increased the risk of having synchronous or metachronous UTUC. However, orifice involvement was not found to be associated with survival outcomes. Development of stricture due to resection is a very rare complication.

## INTRODUCTION

Urothelial carcinoma of the bladder is the most common malignancy of the urinary tract (1, 2). Bladder cancer (BC) may be localized anywhere in the bladder and involvement of ureteral orifice or its close environment has been reported in 5-35% of the cases (3–7). Involvement of ureteral orifice is a diagnostic and therapeutic dilemma as the disease location itself or the applied treatments may cause oncological and functional derangements in the upper urinary tract (5, 6, 8, 9).

Transurethral resection (TUR) of the ureteral orifice is necessary during treatment of these cases (3, 4, 8, 10) and TUR of the ureteral orifice is suggested to cause vesicoureteral reflux (VUR), due to the destruction of the muscle fibers, which leads to upper tract urothelial carcinoma (UTUC) development (5, 8, 9). Additionally, the electro—resection of the ureteral orifice may cause orifice stenosis, and secondary obstruction of the upper urinary tract as well (3, 6, 11).

In the current literature there are a number of studies that report the treatment outcomes of patients with involvement of the ureteral orifice (3–12). These studies involve either relatively low number of patients (6, 8–11) or insufficient follow—up data (3, 5, 7, 8).

In this study, we investigated the data of 138 patients underwent orifice resection from a cohort of 1359 patients underwent TUR for urothelial carcinoma and aimed to report the oncological and physiological outcomes of the patients underwent TUR of the ureteral orifice in comparison with patients that have no evidence of ureteral involvement.

## MATERIALS AND METHODS

This study began after Local Ethics Committee approval, and the medical records were based on the Oncologic Urology Clinics of Tepecik Research and Education Hospital in Izmir, in Turkey. All patients, diagnosed with BC between 1992 and 2012 were reviewed retrospectively, and 1359 patients with available data about tumor localization were evaluated.

The tumors were staged and graded according to the International Union Against Cancer TNM classification and WHO 1973 grading scheme (1, 13). The tumors were classified as <3cm or ≥3cm, and as solitary or multiple. An atrophic kidney was detected in some patients due to obstruction; therefore, the development of hydronephrosis was described as hydronephrosis±atrophic kidney. Tumors involving the ureteral orifice were treated with wide, deep resection, including the entire orifice area, as mentioned in the literature (7, 9, 10). During TUR, pure cutting current was used and selective coagulation was performed to achieve hemostasis. According to our departmental policy, ureteral stenting was avoided. All patients were routinely evaluated via intravenous urography or ultrasound during the first visit and, if necessary, computed tomography and further imaging were performed. During the follow-up, adjuvant intravesical chemotherapy or immuno-therapy, re-TUR, second TUR, imaging, advanced therapy, etc. were performed according to the valid guidelines at the time (13, 14). Survival was calculated from the date of surgery, to either the last follow-up or death.

Statistical analysis was performed using the SPSS 22.0 software program for Windows (SPPS Inc., Chicago, IL, USA). Descriptive statistics for the clinical, pathological and treatment related data were provided. The Student t and Fisher exact tests were used to compare continuous and categorical variables, respectively. Logistic regression analysis was performed to define factors associated with the development of UTUC. Kaplan-Meier analysis was performed to evaluate cancer-specific and overall survival rates of patients with and without ureteral orifice involvement. Cox regression analysis was performed to define the factors associated with survival rates. For statistical significance p-value of 0.05 was accepted.

## RESULTS

Among 1359 patients, 138 (10.2%) had BC involving the ureteral orifice. The two groups did not show significant difference in terms of demographic and cancer-related characteristics except, multiple tumors were significantly more frequent in patients without orifice involvement, and hydronephrosis at the initial diagnosis was more prevalent in the group of patients with orifice involvement. The patients and tumor characteristics are summarized in Table-1. One patient had a history of nephrectomy for renal cell cancer (RCC) before the diagnosis of BC.

**Table 1:** Patients and tumor characteristics.

Characteristics		Orifice involved (n=138)	Non-Orifice involved (n=1221)	Total (n=1359)	P
**Age (Mean ± SD)**		65.1±10.5	63.4±11.7	63.5±11.6	0.087
**Follow-up, months (mean ± IQR)** *		45.5 (9–68)	47.1 (9–70)	46.9 (9–69)	0.721
**No. Gender (%)**					
	M	119 (86.2)	1095 (89.7)	1214 (89.3)	0.214
	F	19 (13.8)	126 (10.3)	145 (10.7)	
**No.TCC tumor grade (%)**					
	G1	47 (34.1)	493 (40.4)	540 (39.7)	
	G2	36 (26.1)	286 (23.4)	322 (23.7)	0.067
	G3	40 (29.0)	248 (20.3)	288 (21.2)	
	Unspecified	15 (10.8)	194 (15.9)	209 (15.4)	
**No.TCC Tumor stage (%)**					
	Ta	47 (34.1)	349 (28.6)	396 (29.1)	
	T1	59 (42.8)	520 (42.6)	579 (42.6)	0.565
	≥T2	32 (23.2)	302 (24.7)	334 (24.6)	
	Unspecified	–	50 (4.1)	50 (3.7)	
**Carsinoma in situ (CIS)(%)**					
	CIS at initial diagnosis	6 (4.3)	42 (3.4)	48 (3.5)	0.584
	CIS progression	3 (2.2)	22 (1.8)	25 (1.8)	0.758
	Total CIS	9 (6.5)	64 (5.2)	73 (5.3)	
**No. Tumor size (%)**					
	Tumor < 3 cm	35 (25.4)	348 (28.5)	383 (28.2)	
	Tumor ≥ 3 cm	94 (68.1)	755 (61.8)	849 (62.5)	0.305
	Unspecified	9 (6.5)	118 (9.7)	127 (9.3)	
**No. Tumor number (%)**					
	Solitary	99 (71.7)	728 (59.6)	827 (60.9)	
	Multiple	36 (26.1)	479 (39.2)	515 (37.9)	0.003
	Unspecified	3 (2.2)	14 (1.1)	17 (1.2)	
**Hydronephrosis (initial diagnosis)(%)**					
	Hydronephrosis±Atrophic kidney	46 (33.3)	170 (13.9)	216 (15.9)	0.0001
**Presence of UTUC (%)**					
	Synchronous	14 (10.1)	8 (0.7)	22 (1.6)	0.0001
	Metachronous*	7 (5.3)	11 (0.9)	18 (1.4)	

* Results of 1299 follow-up patients (132 orifice involved bladder cancer).

### UTUC development

**Figure 1:**
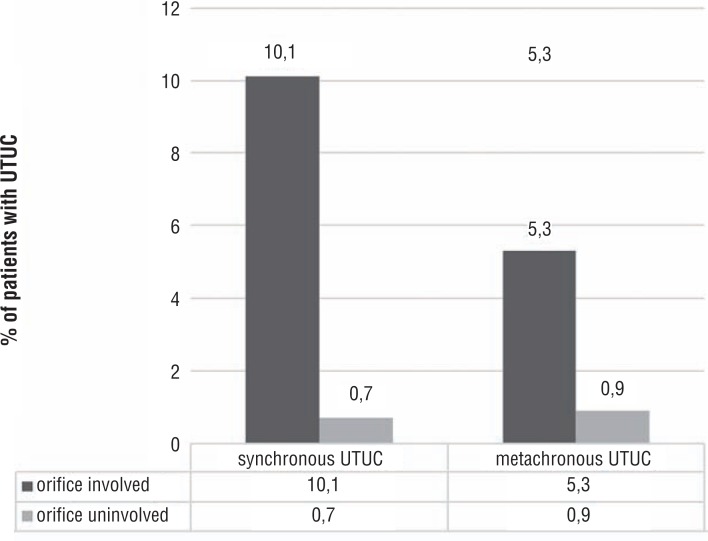
UTUC status.

UTUC was present at the time of diagnosis in 14 of the 138 patients (10.1%) and 8 of 1221 patients (0.7%) in the orifice involved and uninvolved groups respectively (p=0.0001). Rate of metachronous UTUC could be evaluated in 1299 patients (132 orifice involved bladder cancer) and after a mean follow-up of 47 (IQR: 9-69) months, metachronous UTUC developed in 5.3% and 0.9% of the patients in the orifice involved and uninvolved groups of patients respectively (p=0.0001). The results of synchronous and metachronous UTUC are summarized in Figure-1. Logistic regression analysis was performed to determine factors associated with synchronous and metachronous UTUC development. Orifice involvement (OR: 16.044, 95% CI: 6.575-39.151, p=0.0001) and tumor stage (OR: 15.516, 95% CI:1.908-126.182, p=0.01) were identified as the parameters associated with synchronous UTUC development. For metachronous UTUC development, orifice involvement (OR: 9.141, 95% CI: 3.104-26.923, p=0.0001) and T stage (OR: 8.892, 95% CI: 1.163-67.978, p=0.035) were detected as significant. The results of logistic regression analysis are summarized in Table-2.

**Table 2:** Results of logistic regression analysis for development of synchronous and metachronous UTUC.

	Synchronous UTUC development			Metachronous UTUC development		
Parameter	OR	95% CI	P value	OR	95% CI	P value
Age	1.006	0.964-1.051	0.780	0.964	0.922-1.009	0.113
Sex (male vs female)	0.774	0.200-2.994	0.710	0.572	0.069-4.721	0.604
Tumor grade	2.089	0.896-4.868	0.088	1.650	0.164-16.585	0.670
Tumor stage	15.516	1.908-126.182	0.01	8.892	1.163-67.978	0.035
Tumor multiplicity	0.523	0.166-4.648	0.269	0.443	0.158-1.240	0.121
Tumor size (<3 cm vs. ≥3 cm)	0.579	0.200-1.677	0.314	1.731	0.585-5.127	0.322
Orifice involvement	16.044	6.575-39.151	0.0001	9.141	3.104-26.923	0.0001

### Survival analysis

**Figure 2:**
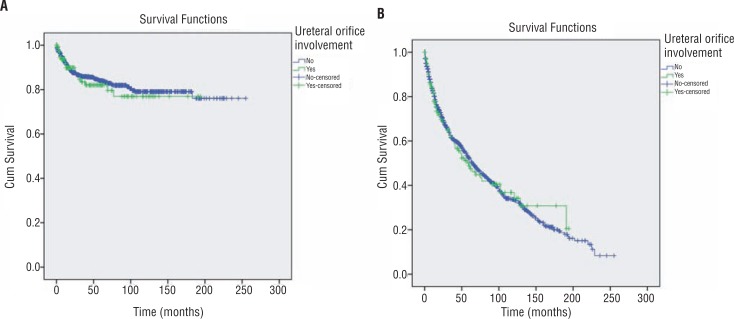
Kaplan-Meier curves for cancer specific (2A) and overall survival (2B).

Kaplan-Meier analysis was performed to determine the effect of orifice involvement on cancer-specific and overall survival. Both cancer-specific and overall survival rates of the orifice involved and uninvolved groups were similar. The survival rates are summarized in Table-3 and Kaplan-Meier figures are given in Figure-2.

**Table 3:** Survival rates of the ureter orifice involved and uninvolved patient groups.

Time	Cancer specific survival rates (%)			Overall survival rates (%)		
	Orifice uninvolved	Orifice involved	P value	Orifice uninvolved	Orifice involved	P value
3 years	85.8	82.0		61.2	60.8	
5 years	83.8	79.6	0.629	52.1	47.5	0.963
10 years	76.1	Not reached		33.4	34.2	

### Functional outcomes

Development of hydronephrosis or renal failure could be evaluated in 132 of the 138 patients with ureteral orifice involvement. One patient underwent nephrectomy due to RCC and hydronephrosis was present in 44 of these patients prior to resection. Seventeen of these 44 patients also had non-functional kidney and 15 of these patients underwent nephrectomy. Hydronephrosis reversed in 10 of the remaining 27 patients (with hydronephrosis and a functioning kidney) after orifice resection. Hydro-nephrosis at the ipsilateral kidney developed in 17 of the 87 remaining patients without initial hydronephrosis. The underlying cause of hydronephrosis was vesicoureteral reflux in 8 (47%) patients, cancer progression and involvement of orifice in 5 (29%) patients, stone disease in 3 (18%) patients and orifice stenosis in 1 (6%) patient.

## DISCUSSION

Involvement of ureteral orifice or its close environment by urothelial carcinoma is observed in up to 35% of the cases (4, 6, 7, 9, 12). Resection of the orifice is necessary in these cases and this has potential to result in loss of anti-reflux mechanism and therefore seeding of malignant cells in the upper urinary tract or ureteral orifice stenosis, which may lead to renal function impairment. In this study, we reported the long-term oncological and functional outcomes of 138 patients underwent ureteral orifice resection due to involvement by urothelial carcinoma and ureteral orifice involvement and resection was shown to increase the risk of UTUC development.

Results of resection of the ureteral orifice have been reported as early as 1936 and in a series of 5 patients, no cases of ureteral orifice stenosis were reported (15). Later on, Rees et al. reported their outcomes in 20 patients, which revealed re-flux in 12 of the 17 patients with follow-up data and no cases of stenosis was reported (4). In these two early series, no data for development of UTUC was available. The first study with evaluation of UTUC development was published by Gottfries et al. and the authors reported their results of 19 patients with a 12 month mean follow-up. In this, no cases of UTUC or ureteral orifice stenosis were reported, with 9 patients found to have reflux (9). Resection of ureteral orifice seems to provide favorable results based on the results of these very early studies which have either very low number of patients of very short duration of follow-up. However, De Torres Mateos et al. reported 26% rate of reflux following resection and they also found a 22-fold greater risk of UTUC development. Therefore, the authors concluded on close follow-up for UTUC development following resection of the ureteral orifice (5). Palou et al. reported the results of their 19 patients underwent resection of the ureter with a mean follow-up of 57 months and they reported UTUC development in 8 patients (42.1%), and nontumoral stenosis in 3 (16%) of the patients. Therefore, the authors also concluded in closer follow-up of the upper urinary tract (11). In a more recent series, Chou et al. reported the results of 31 patients underwent ureteral orifice resection and UTUC was observed in 4 (12.9%) of the patients after a mean follow-up of 33.5 months. Orifice stenosis was reported in 3 (10%) patients as well (6). In another recent series, Mano et al. reported results from 79 patients and 89 renal units underwent ureteral orifice resection. The median follow-up duration was 15 months and they reported 11 (13%) patients to develop hydronephrosis. However, orifice stricture was the cause of hydronephrosis in only 3 (4%) of these patients. UTUC development during the follow-up was reported in only one patient (3).

Our study included a high number of patients with ureteral orifice involvement and different from the previous studies we reported synchronous and metachronous UTUC development separately. Ureteral orifice involvement was found to be associated with 14.4 and 5.7 times increased risk of development of synchronous and metachronous UTUC, respectively. This increased rate of development of metachronous UTUC is parallel to the findings of De Torres Mateos et al. (5). But it is much higher compared to the results of Mano et al. (3), which reported UTUC development in only one patient. This difference may be associated with differences in the duration of follow-up.

Also, the logistic regression analysis revealed ureteral orifice involvement as a significant factor for the development of synchronous and metachronous UTUC. The risk factors for UTUC in primary BC are strongly related to the primary tumor risk stratification, where the incidence is as low as 0.7% in the low-risk group, to as high as 24% in high-risk groups (16). Tumor grade, the presence of carcinoma in situ (CIS), tumor stage, and tumor multiplicity were the factors identified to have an association with the development of UTUC (11, 16, 17). Our result revealed an evidence for the significance of ureteral orifice involvement for further development of UTUC.

This increased risk of UTUC in our population also takes into mind the question of the effect of ureteral orifice involvement on survival rates. Therefore, we performed survival analysis and no significant difference in overall and cancer-specific survival rates were detected between patients with and without ureteral orifice involvement.

Our data indicate that resection of the ureteral orifice resulted in resolution of hydro-nephrosis in 10 of the 27 patients that have hydronephrosis prior to resection. New developed hydronephrosis was observed in 17 of the 87 patients without prior hydronephrosis and orifice stenosis was the cause in only one patient. This result is consistent with the results of the study by Mano et al. (3). In our series, ureteral catheterization following resection was not performed in any of the patients and ureteral stricture developed in only one patient. Therefore, we support the idea of ureteral stenting unnecessary, contrary to the results of the study by Chou et al. which reported 10% obstruction rate (6). Ureteral stenting may be beneficial to prevent the consequences related to ureteral orifice edema, but fibrotic changes were shown to develop after about a month following surgery, which corresponds to the time for extraction of the ureteral stent (18). Therefore, we recommend against routine ureteral stenting following ureteral orifice resection and any symptom related to ureteral orifice edema should be tried to be managed conservatively in the first step.

Our study has some limitations. First of all, retrospective nature and inclusion of patients from a 20 years of time interval limits the homogeneity of follow-up and imaging protocols. Additionally, treatment guidelines showed significant changes during the study period, therefore patients received different adjuvant treatments for urothelial cancer, which has an effect on the survival rates as well.

## CONCLUSIONS

The involvement of the ureteral orifice seems to be an important risk factor for both synchronous metachronous UTUC development. However, ureteral orifice involvement was not found to be associated with overall and cancer specific survival outcomes. Resection of ureteral orifice seems to ahieve acceptable functional outcome results. Clinicians should suspect UTUC in patients with BC involving the ureteral orifice, especially when associated with hydronephrosis.

## Abbreviations

BC: = Bladder Cancer
CIS: = Carcinoma in situ
TUR: = Transurethral resection
UTUC: = Upper tract urothelial carcinoma
VUR: = Vesicoureteral reflux
RCC: = Renal Cell Cancer
